# The uncoupling protein 1 gene, *UCP1*, is expressed in mammalian islet cells and associated with acute insulin response to glucose in African American families from the IRAS Family Study

**DOI:** 10.1186/1472-6823-7-1

**Published:** 2007-03-30

**Authors:** Michèle M Sale, Fang-Chi Hsu, Nicholette D Palmer, Candace J Gordon, Keith L Keene, Hermina M Borgerink, Arun J Sharma, Richard N Bergman, Kent D Taylor, Mohammed F Saad, Jill M Norris

**Affiliations:** 1 Center for Human Genomics, Wake Forest University School of Medicine, Winston-Salem, USA; 2 Department of Internal Medicine, Wake Forest University School of Medicine, Winston-Salem, USA; 3 Center for Public Health Genomics and Department of Medicine, University of Virginia, Charlottesville, USA; 4 Department of Biostatistical Sciences, Wake Forest University School of Medicine, Winston-Salem, USA; 5 Department of Biochemistry, Wake Forest University School of Medicine, Winston-Salem, USA; 6 Department of Comparative Medicine, Wake Forest University School of Medicine, Winston-Salem, USA; 7 Joslin Diabetes Center and Harvard Medical School, Boston, USA; 8 Department of Physiology and Biophysics, Keck School of Medicine, University of Southern California School of Medicine, Los Angeles, USA; 9 Medical Genetics Institute, and Division of Cardiology, Department of Medicine, Cedars-Sinai Medical Center, Los Angeles, USA; 10 Department of Preventive Medicine, Stony Brook School of Medicine, Stony Brook, USA; 11 Department of Preventive Medicine and Biometrics, University of Colorado Health Sciences Center, Denver, USA

## Abstract

**Background:**

Variants of uncoupling protein genes *UCP1 *and *UCP2 *have been associated with a range of traits. We wished to evaluate contributions of known *UCP1 *and *UCP2 *variants to metabolic traits in the Insulin Resistance and Atherosclerosis (IRAS) Family Study.

**Methods:**

We genotyped five promoter or coding single nucleotide polymorphisms (SNPs) in 239 African American (AA) participants and 583 Hispanic participants from San Antonio (SA) and San Luis Valley. Generalized estimating equations using a sandwich estimator of the variance and exchangeable correlation to account for familial correlation were computed for the test of genotypic association, and dominant, additive and recessive models. Tests were adjusted for age, gender and BMI (glucose homeostasis and lipid traits), or age and gender (obesity traits), and empirical P-values estimated using a gene dropping approach.

**Results:**

*UCP1 *A-3826G was associated with AIR_g _in AA (P = 0.006) and approached significance in Hispanic families (P = 0.054); and with HDL-C levels in SA families (P = 0.0004). Although *UCP1 *expression is reported to be restricted to adipose tissue, RT-PCR indicated that *UCP1 *is expressed in human pancreas and MIN-6 cells, and immunohistochemistry demonstrated co-localization of UCP1 protein with insulin in human islets. *UCP2 *A55V was associated with waist circumference (P = 0.045) in AA, and BMI in SA (P = 0.018); and *UCP2 *G-866A with waist-to-hip ratio in AA (P = 0.016).

**Conclusion:**

This study suggests a functional variant of *UCP1 *contributes to the variance of AIR_g _in an AA population; the plausibility of this unexpected association is supported by the novel finding that *UCP1 *is expressed in islets.

## Background

Uncoupling proteins are mitochondrial inner membrane electron carriers [1]. Uncoupling protein 2 (UCP2) inhibits glucose stimulated insulin secretion, and models of type 2 diabetes are associated with increased expression in islets [2]. Polymorphisms in the uncoupling protein genes *UCP1 *(4q31.21) and *UCP2 *(11q13.4) have been associated with a number of measures of glucose homeostasis and adiposity. These include associations between *UCP2 *G866A and A55V and glucose-induced insulin secretion [3-5], as well as obesity and metabolism [6-8]. Associations with obesity, weight gain and metabolism [9-12] have been reported for *UCP1 *variant A3826G, often acting synergistically with β3-adrenergic receptor W64R, and *UCP1 *A64T has also been associated with obesity [10]. In addition, *UCP1 *A-3826G and M229L have been found to be associated with type 2 diabetes [11,13].

Given the importance of *UCP1 *and *UCP2 *genes in metabolism, we chose to evaluate the five promoter or coding variants in these two genes previously associated with metabolic phenotypes (above) for associations in families from the Insulin Resistance and Atherosclerosis (IRAS) Family Study. The IRAS Family Study has recruited extended African American and Hispanic families [14], and extensive phenotypic data on measures of glucose homeostasis, adiposity, and lipids have been collected on participants. On detection of a significant association between *UCP1 *SNPs and acute insulin response to glucose (AIR_g_), we investigated the expression patterns of this gene in mammalian pancreas.

## Methods

### Subjects

The IRAS Family Study design, recruitment and phenotyping have been described in detail [14]. Studies were conducted using protocols approved by the human subjects committees at each participating institution and all participants provided informed consent. Briefly, multi-generational African American and Hispanic families were initially recruited from probands of the original IRAS cohort [15]. Ascertainment of the proband was based on the sample size of available family members (with a target of four living full siblings and five living offspring of these siblings) and a range of glucose tolerance. Ascertainment was supplemented with additional large non-IRAS families recruited from the general population. Families were not selected based on any phenotypic criteria. Participants were from clinical centers in Los Angeles, California (African American), San Luis Valley, Colorado (rural Hispanic), and San Antonio, Texas (urban Hispanic). The clinical examination included height, weight, waist and hip circumferences, fasting blood draw, computerized tomography (CT) scanning for assessment of abdominal fat area, and medical history interview. A total of 287 African American individuals (18 families; family size ranged from 4 to 51 family members) from Los Angeles, 318 Hispanic individuals (12 families; family size 4 to 39 members) from San Luis Valley and 493 Hispanic individuals (33 families; family size from 6 to 30 members) from San Antonio were included in the analyses (Table 1).

**Table 1:** IRAS Family Study participant characteristics

	**African American**	**Hispanic**	
	**Los Angeles**	**San Luis Valley**	**San Antonio**
Families (n)	18	12	33
Family members per family (n)	4–51	4–39	6–30
Participants^a ^(n)	287	318	493
Female gender, %	56.5	52.2	60.0
Diabetes, %	11.6	12.8	17.7
Age, mean ± SD	43.8 ± 14.8	40.3 ± 14.0	43.6 ± 14.8
AIR_g _μU/mL ± SD	878.4 ± 768.2	808.1 ± 631.5	726.2 ± 620.1
S_I_, MINMOD, ± SD	1.75 ± 1.22	2.41 ± 2.00	1.95 ± 1.85
BMI, kg/m^2 ^± SD	28.8 ± 6.5	27.5 ± 5.6	30.1 ± 6.3
Waist, cm ± SD	89.3 ± 14.1	87.3 ± 13.2	92.9 ± 14.7
WHR, ratio ± SD	0.82 ± 0.08	0.85 ± 0.09	0.85 ± 0.08
HDL, mg/dL ± SD	48.3 ± 13.2	43.7 ± 12.4	42.4 ± 12.9

^a^Includes only individuals from the IRAS Family Study cohort with at least one *UCP1 *or *UCP2 *SNP genotype. Individuals in these families with type 2 diabetes have been excluded from AIR_g _and S_I _calculations. AIR_g_: Acute insulin response to glucose; S_I_: insulin sensitivity index; WHR: waist-to-hip ratio.

### Glucose homeostasis traits

The following traits related to glucose homeostasis were tested for association with the *UCP1 *and *UCP2 *SNPs genotyped: fasting plasma glucose; fasting plasma insulin; acute insulin response to glucose (AIR_g_); and insulin resistance, expressed as the insulin sensitivity index (S_I_). Glucose values were obtained after a minimum 8 hour fast. Plasma glucose and insulin levels were measured at the University of Southern California, using the glucose oxidase technique on an autoanalyzer and the insulin dextran-charcoal immunoassay [16]. Insulin sensitivity was assessed by the frequently sampled intravenous glucose tolerance test (FSIGT), using a reduced sampling protocol [17]. Glucose in the form of a 50% solution (0.3 g/kg) and regular human insulin (0.03 μ/kg) were injected through an intravenous line at 0 and 20 min, respectively. Blood was collected at -5, 2, 4, 8, 19, 22, 30, 40, 50, 70, 100, and 180 min. Insulin resistance, expressed as the insulin sensitivity index (S_I_), was calculated by mathematical modeling methods (MINMOD) [18]. Acute insulin response to glucose (AIR_g_) was defined as the mean insulin increment in the plasma insulin concentration above the basal in the first 8 min after the administration of glucose. Glucose values were obtained after a minimum 8 hour fast, and diabetes was diagnosed using the American Diabetes Association criteria of fasting plasma glucose value ≥ 126 mg/dL and/or current use of anti-diabetic medications. Individuals with diabetes were excluded for analyses of glucose homeostasis traits. One hundred eighty-five participants had impaired fasting glycemia (fasting glucose > 100 mg/dL).

### Lipid traits

Lipid traits tested for association with *UCP1 *and *UCP2 *SNPs included: triglyceride; HDL-C; LDL-C; and total cholesterol levels. Plasma was separated from blood collected after a 12 h fast, and stored at -70°C prior to analysis. Total cholesterol and triglyceride were measured using enzymatic methods. LDL-C was calculated using the Friedewald equation [19] if triglyceride was < 400 mg/dL or otherwise by ultracentrifugation. HDL-C was measured using the direct method [20].

### Obesity and adiposity traits

Obesity traits used for association analyses included: waist circumference; waist-to-hip ratio (WHR); BMI; visceral adipose tissue (VAT); and subcutaneous adipose tissue (SAT). Height, and waist and hip circumferences were measured to the nearest 0.5 cm, and weight to the nearest 0.1 kg. BMI was calculated as weight (kg)/height (m)^2^. Abdominal fat mass was measured at the L2/L3 and L4/L5 vertebral region by CT. Scans were read at the University of Colorado Health Sciences Center, Department of Radiology, for VAT and SAT. Bowel fat was subtracted out from the VAT, and L4/L5 measures used in these analyses. A small number of participants were missing L4/L5 data but had L2/L3 data; for these participants L4/L5 data was imputed from the L2/L3 data using a simple linear model.

### UCP1 and UCP2 genotyping

Total genomic DNA was purified from whole blood using the PUREGENE DNA Purification kit (Gentra Systems, Minneapolis MN). Genotyping was conducted using a MassARRAY system (Sequenom, San Diego CA) [21]. Primer sequences for *UCP1 *A-3826G, A64T, M229L, and *UCP2 *G-866A and A55V, designed using SpectroDESIGNER software (Sequenom, San Diego CA), are available on request.

### Statistical analyses

#### Relationship testing and genotyping error checking

As part of the broader IRAS Family Study efforts, pedigrees were genotyped by the Mammalian Genotyping Service (MGS), Marshfield WI, using 383 microsatellite markers. Each pedigree was examined for consistency of the stated family structure with the genome scan data using PREST, version 2.01 [22,23]. In the 63 pedigrees used in this investigation, a total of 28 likely misspecified familial relationships were modified from 18 families. Genotypes that showed inconsistency with Mendelian inheritance were identified using PedCheck, version 1.1 [24], and inconsistent genotypes converted to missing. All SNPs were checked for consistency with Hardy Weinberg Equilibrium (HWE).

#### Linkage disequilibrium

We estimated the degree of linkage disequilibrium (LD) between SNPs using the standardized measure of D' [25], where the joint probability of haplotype was estimated by the expectation-maximization (EM) algorithm and the respective allele frequencies were estimated based on maximum likelihood estimates [26].

#### Association analyses

To explore associations among SNP polymorphisms and quantitative measures, the marginal models incorporating generalized estimating equations (GEE1) [27] were used. This approach has been widely used in longitudinal data analysis [28] and can account for the dependency between correlated measures within a family. The method allows separate modeling of the regression of quantitative measures on SNP polymorphisms and other covariates, and the association among quantitative measures within each family. An advantage of this method is that one need not to specify the distribution of the outcome variable, just the relationships between the marginal mean and variance, and between the marginal mean and covariates. Another advantage is that even though the correlation model among the related outcome measures may be specified incorrectly, the association model between the outcome and covariates can still obtain a robust result. Here familial correlation was accounted for using a sandwich estimator of the variance and exchangeable correlation. A family of power transformations conditional on the covariates age, gender, and BMI [29] was explored. To minimize the heterogeneity of variance, the phenotypes were transformed to best approximate the normality assumptions. We computed four tests of association for each SNP: the overall test of genotypic association with two degrees of freedom, as well as the statistical contrasts defined by three genetic models – dominant, additive, and recessive. Age, gender and BMI were included as covariates, except for measures of adiposity where analyses were conducted with and without BMI as a covariate. Marginal regression coefficients have the same interpretation as those from an analysis using unrelated individuals. Potential influential points and outliers have been checked. The analyses were stratified by center and performed using SAS software (SAS Institute, Cary, NC).

To avoid the potential increase of type I error rate, we estimated empirical pvalues for significant single SNP GEE association analyses (P < 0.05 using the general model, with mode of inheritance subsequently explored). We used the gene dropping approach implemented in Mendel, version 5.7 [30], to simulate 10,000 datasets based on the IRAS Family Study pedigree structure under the null hypothesis of no association between phenotype and genotype data. The empirical p-value was determined as the proportion of simulated data sets with statistics more extreme than the observed value. Only empirical p-values are presented since this approach is more conservative and adjusts for deficiencies in the large sample approximation of the GEE method.

Two-marker (*UCP1 *and *UCP2*) and three-marker (*UCP1 *only) haplotypic associations were evaluated in each population separately using quantitative pedigree disequilibrium tests (QPDT) [31].

#### RT-PCR

Reverse Transcription-PCR (RT-PCR) was performed using the SuperScript First Strand Synthesis System for RT-PCR (Invitrogen Life Technologies, Carlsbad, CA), as described by the manufacturer, using 1 μg total human pancreas RNA (Ambion, Austin, TX). Two μl cDNA were subsequently amplified in a total reaction volume of 50 μl containing 0.2 mM dNTP, 1.5 mM MgCl_2_, and 200 nM of each forward and reverse primer. Primers were specifically designed in regions of low homology with the other *UCP *genes and flanking one or more introns. For human *UCP1 *the following primers were used: forward primer 5'-TGGAATAGCGGCGTGCTTG-3' (located in exon 1), and reverse primer 5'-CTCATCAGATTGGGAGTAG-3' (exon 4), expected to produce a product of 489 bp. For human *UCP2*, these primers were used: forward 5'-TCTACAATGGGCTGGTTGC-3' (exon 2) and reverse 5'-TGTATCTCGTCTTGACCAC-3' (exon 5), with an expected product size of 495 bp. Control primers were for β-actin, supplied in the SuperScript First Strand Synthesis System for RT-PCR (Invitrogen Life Technologies, Carlsbad, CA). Each reaction was denatured at 95°C for 2 minutes, amplified using 40 cycles of 95°C for 30 seconds, 60°C for 45 seconds, and 72°C for 30 seconds, and underwent a final extension at 72°C for 3 minutes. Samples were electrophoresed on a 1% agarose gel, products visualized under UV light, and compared against a Ready-Load 1 Kb Plus DNA Ladder (Invitrogen, Carlsbad, CA). Bands were extracted using a QIAquick Gel Extraction Kit microcentrifuge protocol (Qiagen Sciences, Valencia, CA) and sequenced on an ABI PRISM 3100 Genetic Analyzer (Applied Biosystems, Foster City, CA) using Big Dye Terminator v3.1 Cycle Sequencing Kits (Applied Biosystems, Foster City, CA).

Total RNA was extracted from MIN-6 or αTC 1.6 cells and reverse-transcribed to cDNA. 10 ng of cDNA was used in a 25 μl PCR reaction with AmpliTaq Gold Polymerase, using the following amplification conditions for *Ucp2*: 95°C for 10 min, Hot-start, then 35 cycles of denaturation at 95°C for 30 sec, annealing at 60°C for 30 sec and amplification at 72°C for 30 sec. The same amount of cDNA was used in 25 μl PCR reaction with AmpliTaq Gold Polymerase to detect *Ucp1*. PCR conditions for *Ucp1 *were: 95°C for 10 min, Hot-start, followed by 3 cycles of denaturation at 95°C for 30 sec, annealing at 54°C for 10 sec, extension at 72°C for 30 sec; another 3 cycles at 95°C for 30 sec, 56°C for 15 sec and 72°C for 30 sec, and finally 35 cycles at 95°C for 30 sec, 58°C for 30 sec and 72°C for 30 sec. After the last cycle, samples were incubated for 5 min at 72°C. 15 μl of amplified products were then resolved on a 1% agarose gel, and DNA bands visualized under UV. Primers used to amplify mouse *Ucp1 *and *Ucp2 *products were UCP1-M F1, 5'-TATCATCACCTTCCCGCTG-3' (exon 1) ; UCP1-M R1, 5'-GTCATATGTTACCAGCTCTG-3' (exon 4), product size 505 bp; UCP2-M F2, 5'-TCTACAATGGGCTGGTCGC-3' (exon 4); UCP2-M R5, 5'-CAAGCGGAGAAAGGAAGGC-3' (exon 8), product size 608 bp. The quality of MIN-6 and αTC1.6 cDNA was confirmed by PCR amplification of cyclophilin (data not shown).

#### Immunohistochemistry

Formalin-fixed, paraffin embedded *Macaca fascicularis *(monkey) pancreas was sectioned at 4 μm, and commercially obtained normal human (BioChain, Haywood, CA) and normal mouse pancreatic tissue sections (BioChain, Haywood, CA) were used. Triplicate slides for each species were used for the localization of (1) insulin, (2) UCP1, and (3) insulin and UCP1. After sections were deparaffinized, two sets of slides were incubated with Guinea pig anti-porcine insulin (DAKO, Carpinteria, CA) at 1:100 for 60 minutes at 34°C, then incubated with biotinylated anti-rabbit at 1:20 (BioGenex, San Ramon, CA) for 10 minutes at 34°C, and finally incubated for 20 minutes at 37°C with Streptavidin-β-Galactosidase at 1:250 (Gibco-BRL, Gaithersburg, MD) and visualized using Gal-X. After performing antigen retrieval, insulin-stained as well as unstained sections were incubated with goat anti-UCP1 (Santa Cruz Biotechnology, Santa Cruz, CA) at 1:100 for one hour at room temperature, incubated 10 minutes at 34°C in biotinylated rabbit anti-goat (Jackson ImmunoResearch, West Grove, PA) at 1:250, then incubated with Streptavidin-Alkaline phosphatase (BioGenex, San Ramon, CA) at 1:20 for 10 minutes at 34°C. Vector Red substrate (Vector Laboratories, Burlingame, CA) was used for visualization, and sections were counterstained with Mayer's hematoxylin. A MicroProbe system (Fisher Scientific, Kent, WA) using capillary action, and commercially purchased TRIS diluent and wash buffer (Biomeda, Foster City, CA) with 0.1% Tween 20 to serve as a surfactant and 0.5% casein to block non-specific binding of proteins were used.

## Results

### Study sample

Characteristics of the three IRAS Family Study populations are shown in Table 1. The mean ages of the participating family members were 43.8 ± 14.8 years (Los Angeles), 40.3 ± 14.0 years (San Luis Valley) and 43.6 ± 14.8 years (San Antonio), with 52.2–60.0% female participants. Using the Los Angeles population as the reference group, the mean age of the San Luis Valley participants is significantly lower than the Los Angeles participants (p = 0.0057). The prevalence of diabetes in the San Antonio participants is higher than in the Los Angeles sample (p = 0.046) and the mean AIR_g _in the San Antonio sample is significantly lower than that of the Los Angeles population (p = 0.024). A higher AIR_g _in African American participants, when compared with Hispanic participants, has been reported previously for this study population [23,32,33]. The mean waist measurement in San Antonio family members (p = 0.043) is significantly greater than that of the Los Angeles participants; and the mean WHR in San Antonio (p = 0.0004) and San Luis Valley (p = 0.0028) samples are significantly higher than that of the Los Angeles sample. The mean HDL in San Antonio (p < 0.0001) and San Luis Valley (p = 0.0057) families are both significantly lower than in the Los Angeles families.

### HWE, linkage disequilibrium between SNPs, and allele frequencies

All SNPs were consistent with HWE, except for *UCP1 *A-3826G in the San Antonio Hispanic population (P = 0.034 for unrelated founder individuals, n = 73). There was a single inconsistency (for *UCP1 *A64T in an individual from the San Luis Valley) among genotypes from 50 individuals included as blind duplicates, producing a genotyping error rate of 0.4% and a concordance rate of > 99%. *UCP1 *SNPs were in high LD, with D' > 0.90 for all pairwise comparisons across all three populations using unrelated founders, except for A64T and M229L in the SA founders where D' = 0.80. LD between the two *UCP2 *SNPs was high, with D' > 0.97 across all three populations. Genotype frequencies are shown in Table 2. *UCP1 *SNPs A64T and M229L were relatively rare, and recessive effects of these SNPs were disregarded in this study.

**Table 2:** Genotype frequencies for *UCP1 *and *UCP2 *SNPs in populations from the IRAS Family Study

			**African American**	**Hispanic**	
			**Los Angeles**	**San Luis Valley**	**San Antonio**
***UCP1***	**A-3826G**	A/A, % (n)*	17.4 (50)	34.0 (108)	35.2 (174)
		A/G, % (n)	40.4 (116)	51.9 (165)	50.4 (249)
		G/G, % (n)	42.2 (121)	14.2 (45)	14.4 (71)
		Total (n)	(287)	(318)	(494)
	**A64T**	G/G, % (n)	83.1 (236)	83.8 (263)	83.1 (409)
		G/A, % (n)	16.2 (46)	15.6 (49)	15.9 (78)
		A/A, % (n)	0.7 (2)	0.6 (2)	1.0 (5)
		Total (n)	(284)	(314)	(492)
	**M229L**	A/A, % (n)	93.0 (264)	84.4 (265)	82.9 (402)
		A/T, % (n)	7.0 (20)	14.7 (46)	16.1 (78)
		T/T, % (n)	0 (0)	1.0 (3)	1.0 (5)
		Total (n)	(284)	(314)	(485)
***UCP2***	**G-866A**	G/G, % (n)	32.8 (89)	31.1 (94)	27.2 (132)
		G/A, % (n)	48.0 (130)	48.7 (147)	46.7 (227)
		A/A, % (n)	19.2 (52)	20.2 (61)	26.1 (127)
		Total (n)	(271)	(302)	(486)
	**A55V**	C/C, % (n)	28.8 (82)	28.3 (89)	26.3 (129)
		C/T, % (n)	51.2 (146)	53.3 (168)	47.4 (232)
		T/T, % (n)	20.0 (57)	18.4 (58)	26.3 (129)
		Total (n)	(285)	(315)	(490)

*n = the number of individuals successfully genotyped.

### Association between UCP1 and AIR_g_

A significant association was observed between *UCP1 *A-3826G and AIR_g _in African American families (GEE empirical P = 0.006, dominant model; Table 3). There was borderline evidence for an association between A-3826G and AIR_g _in Hispanic families from San Luis Valley, Colorado (GEE empirical P = 0.054, dominant model). Although the initial GEE analysis provided evidence of an association between A64T and AIR_g _in African Americans (unadjusted p < 0.001, general model; P = 0.038, additive model), this result was driven by the presence of two minor allele homozygotes with high AIR_g _values and was not supported by simulation-based empirical p-values (P = 0.116, general model; P = 0.110, additive model). Haplotypic association analyses of these two SNPs using QPDT [31] were not significant (data not shown).

**Table 3:** Significant association results between *UCP1 *and *UCP2 *SNPs and traits in populations from the IRAS Family Study

**Trait**	**Population**	**Gene**	**SNP**	**Allele 1/Allele 2**	**Mean ± SE 1/1 (n)**	**Mean ± SE 1/2 (n)**	**Mean ± SE 2/2 (n)**	**P-value (General)**	**P-value (Dom)**	**P-Value (Add)**	**P-value (Rec)**
AIR_g_^a^	Los Angeles	*UCP1*	A3826G	A/G	669.4 ± 86.3 (42)	954.2 ± 83.9 (93)	894.7 ± 65.1 (98)	**0.017**	**0.006**	0.366	0.786
AIR_g_^a^	San Luis Valley	*UCP1*	A3826G	A/G	718.4 ± 110.5 (74)	867.3 ± 81.9 (117)	855.9 ± 80.2 (23)	0.161	0.054	0.093	0.366
BMI^b^	San Antonio	*UCP2*	A55V	C/T	29.2 ± 0.82 (115)	28.7 ± 0.54 (210)	30.3 ± 0.71 (116)	**0.040**	0.968	0.220	**0.018**
Waist^b^	Los Angeles	*UCP2*	A55V	C/T	88.1 ± 0.60 (76)	87.1 ± 0.49 (139)	89.4 ± 0.67 (54)	**0.045**	0.440	0.510	0.065
WHR^b^	Los Angeles	*UCP2*	G-866A	G/A	0.82 ± 0.005 (77)	0.81 ± 0.005 (130)	0.83 ± 0.007 (51)	**0.016**	0.237	0.863	**0.037**
HDL^a^	San Antonio	*UCP1*	A3826G	A/G	40.2 ± 1.14 (164)	42.8 ± 1.10 (217)	44.9 ± 1.46 (66)	**0.001**	**0.015**	**0.0004**	**0.006**

Empirical P-values are shown. ^a^Adjusted for age, sex and BMI; ^b^adjusted for age and sex. General: General model; Dom: Dominant model; Add: Additive model; Rec: Recessive model. **Bold**: P < 0.05.

### Other significant associations

Four other significant associations were detected (Table 3), with the majority related to measures of adiposity. *UCP2 *A55V was associated with waist circumference (P = 0.045) and *UCP2 *G-866A with WHR in AA (P = 0.016), although only in analyses unadjusted for BMI (but adjusted for age and sex). *UCP2 *A55V was also associated with BMI in SA (P = 0.018), and *UCP1 *A-3826G was associated with HDL-C levels in SA families (P = 0.0004) after adjustment for age and sex. Even though associations with *UCP2 *A55V were across different populations, the direction of effects was consistent, with the 55V/55V genotype associated with both greater waist circumference and higher BMI.

### RT-PCR of UCP1 and UCP2

**Figure 1:**
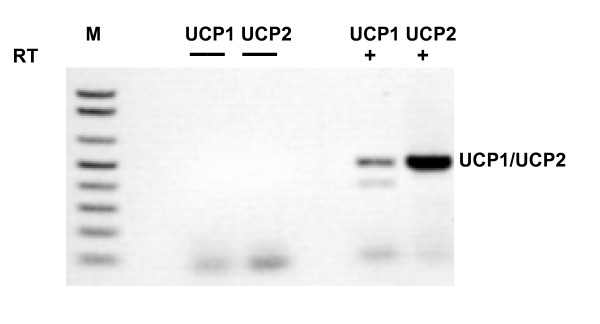
UCP1 is expressed in human total pancreatic RNA: cDNAs prepared in the absence or presence of reverse transcriptase (RT) from total human pancreas RNA, were used for PCR with the indicated primers. The lane containing 1 Kb Plus DNA Ladder (M), and positions of *UCP1 *(489 bp) and *UCP2 *products (495 bp) in the lanes corresponding to RT+ cDNA are indicated.

**Figure 2:**
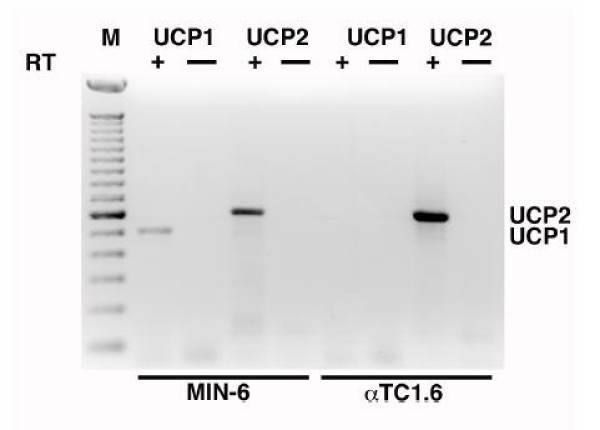
*Ucp1 *is expressed in an insulin producing cell line: cDNAs prepared in the absence or presence of reverse transcriptase (RT) from total RNA isolated from MIN-6 and αTC1.6 cells, were used for PCR with the indicated primers. The lane containing 100 bp marker (M), and positions of *Ucp1 *and *Ucp2 *products are indicated. A band corresponding to *Ucp1 *(505 bp) can be clearly seen in the lane corresponding to RT+ MIN-6 cDNA. Expression of *Ucp2 *(608 bp) can be seen in both insulin and glucagon producing cell lines.

Results of the RT-PCR analyses using total human pancreatic RNA are shown in Figure 1. There was no product visible when the protocol was carried out without the addition of RT enzyme (-RT control; Figure 1, lanes 3 and 4), indicating no detectable contamination with genomic DNA. A band representing the *UCP1 *product (489 kb) can be seen in Figure 1, lane 6. The identity of this band as the expected *UCP1 *product was confirmed by sequencing. A second spurious band is also seen in lane 6 at 381 bp. This product was also sequenced and BLAST searches [34] indicated 98% homology to a region of the *SAR1 *gene on 10q22.1. There is only 13% homology between this product and the *UCP1 *product, however there is an 8-base region in the *UCP1 *forward primer and a 6-base sequence in the *UCP1 *reverse primer that are identical to regions in the *SAR1 *gene. These sequences immediately flank the *SAR1*-like sequence obtained and may have served as an anchor to allow amplification. We never detected *UCP1 *expression in the absence of the *SAR1 *band. As expected, a 495 kb band derived from *UCP2 *is present in Figure 1, lane 7 and its origin was also confirmed by sequencing. Expression of *UCP1 *was also detected at low levels in the murine beta cell line MIN-6 (Figure 2, lane 2), but was absent in the murine glucagon-secreting cell line αTC1.6 (Figure 2, lane 6).

### Immunohistochemistry of pancreatic sections

**Figure 3:**
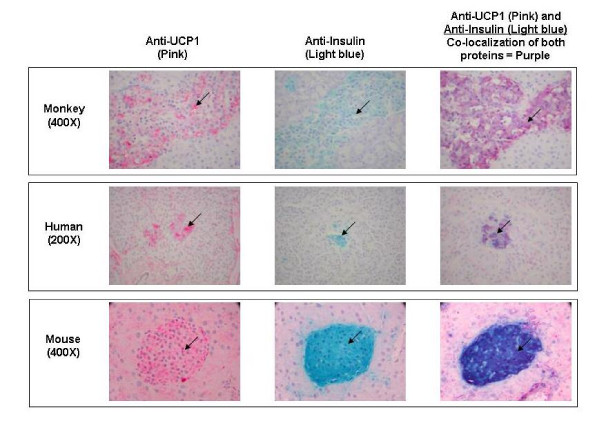
UCP1 and insulin co-localize in mammalian islet cells. Pancreas sections from monkey (*Macaca fascicularis*), human, and mouse (indicated at left, together with magnification) were stained using immunohistochemistry with anti-UCP1 (left); anti-Insulin (middle); or double-stained with anti-UCP1 and anti-Insulin (right). Sections stained with anti-UCP1 were visualized with Vector Red, resulting in pink staining of the islet cells, seen in the left panel for each species. Gal-X visualization of anti-Insulin produced a light blue stain of islet cell cytoplasm, seen in the middle panel for each species. Co-localization (overlay) of both anti-UCP1 (pink) and anti-Insulin (light blue) stains produced the purple staining of the islets seen in the third panel for each species. Examples of islet cells with cytoplasmic staining for the proteins of interest are indicated by the arrows.

Results of the IHC experiments are shown in Figure 3. UCP1 protein was detected in pancreatic tissue sections from *Macaca fascicularis *(monkey), human, and mouse, and co-localized with insulin in the islet cells of all three species. As anticipated from its known mitochondrial localization, UCP1 protein was only observed in the cytosol.

## Discussion

As anticipated, associations were detected between genotyped SNPs and metabolic traits, particularly with measures related to adiposity: *UCP2 *SNPs with waist circumference, BMI and waist-to-hip ratio, although significance levels for these associations were modest (Table 3). An association between *UCP1 *A-3826G and HDL-C levels in SA families (P = 0.0004) was also detected. The association between *UCP1 *A-3826G and AIR_g _in the AA families from the IRAS Family Study was unexpected, since *UCP1 *has not been reported to be expressed in the pancreas.

In both the original IRAS cohort [35], and the IRAS Family Study [32], AIR_g _was found to be higher in non-diabetic African Americans than non-Hispanic whites or Hispanics. Carrying either the *UCP1 *3826G allele or the rare 64T allele increases AIR_g _(although the latter association was not statistically significant). The higher frequency of 3826G in the African American families investigated appears to at least partially explain the higher AIR_g _values. We did not find significant association with AIR_g _using haplotype analysis of the two *UCP1 *SNPs, and there is insufficient power in the present study design to investigate whether the presence of the two SNPs in *trans *show an interaction. Interestingly, threonine is present at position 64 in dog, mouse, rat, hamster, rabbit, *Arabidopsis *and *Solanum *Ucp1, and also present in human UCP2 and UCP3, although alanine is the more common amino acid in UCP1 of humans. It is not known what effect substituting a hydrophilic amino acid (threonine) for a hydrophobic residue (alanine) at this position has on protein function, especially in relation to insulin secretion.

While *UCP2 *is expressed in a variety of tissues [36], *UCP1 *expression was believed to be largely restricted to brown adipose tissue (BAT) [36-38]. However, expression of *ucp1 *in mouse brain, skeletal muscle [39] and uterus [40], and rat thymus [41] have been reported. The rapid insulin response measured by AIR_g _is generally believed to be a measure of beta cell function, and we therefore sought to explain the biological impact of *UCP1 *on AIR_g _by investigating the expression of this gene in the pancreas. Detection of *UCP1 *expression in human pancreas by RT-PCR is a novel finding. We confirmed the presence of UCP1 in human pancreas by IHC and found the UCP1 protein co-localized with insulin in the islets. Further, RT-PCR of the murine beta cell line MIN-6 demonstrated the presence of *UCP1 *expression in beta cells, while *UCP1 *mRNA could not be detected in the murine alpha cell derived line αTC1.6.

*UCP2 *is known to be expressed in pancreatic islets, where it is upregulated by glucolipotoxic conditions, and increased *UCP2 *expression decreases glucose-stimulated insulin secretion [42,43]. Even though *UCP2 *G866A has been reported by others as influencing glucose-induced insulin secretion [3-5], we did not see any association between this SNP and AIR_g_. Over-expression studies of *ucp1 *in INS-1 cells have shown that *ucp1 *upregulation also suppresses insulin secretion [44], while studies of adipose tissues indicated that the promoter -3826G allele decreases *UCP1 *expression [45]. Assuming the same impact on expression is true in human beta cells, this would be consistent with the increased first-phase insulin response (or lack of suppression) seen individuals from Los Angeles and San Luis Valley with one or two copies of the *UCP1 *-3826G allele.

The polygenic regulation of AIR_g_, and implication from expression studies that other sequence variants are likely to influence *UCP1 *expression [45], may explain the more modest effects (San Luis Valley) or lack of association (San Antonio) seen between *UCP1 *A-3826G and AIR_g _in the two Hispanic populations.

Associations between *UCP2 *SNPs and waist or WHR were modest and have not been reported previously, although *UCP2 *G-866A and A55V have been reported as associated with BMI [46-48], and G-866A associated with obesity [8] and adipose tissue levels [4]. The -866A allele is associated with enhanced *UCP2 *mRNA expression in adipose tissue [8], possibly via *PAX6 *transactivation [49]. However, commonly reported associations between obesity and the *UCP1 *SNPs genotyped were not detected. The reported association between *UCP1 *A-3826G and obesity in Spanish women [50] was not observed in Hispanic families, possibly due to different environmental interactions. The smallest P-value for this SNP with the 2 degree-of freedom general test of association and measures of obesity was P = 0.14 for BMI in San Luis Valley women.

Interestingly, the same *UCP1 *SNP associated with high AIR_g _in AA, 3826G, was also significantly associated with higher HDL-C levels in the Hispanic SA families, although it should be noted that this SNP deviated from HW proportions (P = 0.034) in the 73 founders in this population. In a study of 312 Japanese women, there was a non-significant trend for women carrying the 3826G allele to have higher HDL levels than non-carriers, in both premenopausal (P = 0.14) and postmenopausal (P = 0.29) groups [51]. However a study of 118 obese Polish subjects showed 3826G/G homozygotes (n = 17) had significantly *lower *levels of HDL-C than AA homozygotes (n = 63) (P = 0.004) [52], opposite to the results observed in the SA families of the IRAS Family Study.

Tests of association for additional traits (such as hypertension and coagulation factors) that were not part of our *a priori *hypotheses for either *UCP1 *or *UCP2 *were conducted during automated analytical procedures. After Bonferroni correction for 5 SNPs × 21 traits × 4 models + 4 haplotypic analyses, none of the reported associations would maintain significance. However, all tests can not be considered entirely independent due to correlations between SNPs and across phenotypes, so this approach may be overly conservative. Additionally, we did not consider the inheritance models unless the general test of association was significant. While a trend between *UCP1 *3826G and higher HDL-C levels detected in the SA population has been reported previously [51], clearly replication of the association between this SNP and AIR_g _in other populations will be needed to confirm this finding.

## Conclusion

We have confirmed the previously-reported association between *UCP1 *A-3826G and HDL-C levels in a Hispanic population from the IRAS Family Study. We also found a novel association between *UCP1 *A-3826G and AIR_g _in an African American population, and detected *UCP1 *expression in primate and mouse islet cells. These results suggest that UCP1 may have a previously unsuspected role in first-phase insulin release. Further studies of the influence of variants of the *UCP1 *gene on AIR_g _in other populations, and investigations of the interplay between UCP1 and UCP2 on glucose-stimulated insulin secretion, are warranted.

## Abbreviations

UCP: uncoupling protein; AIR_g_: acute insulin response to glucose; QPDT: quantitative pedigree disequilibrium test; HWE: Hardy Weinberg equilibrium

## Competing interests

The author(s) declare that they have no competing interests.

## Authors' contributions

MMS reviewed association results, planned expression and immunohistochemistry experiments, and drafted the manuscript. FCH conducted statistical analyses. NDP selected SNPs and performed genotyping assays. CJG and KLK conducted RT-PCR and sequencing assays of human pancreas, while RT-PCR of MIN-6 and αTC1.6 cells were provided by AJS. HMB conducted the immunohistochemistry experiments. RNB, MFS, and JMN were involved in the conception, design and phenotypic characterization of the IRAS Family Study. MFS and JMN also played a key role in recruitment, and KDT contributed to genotypic characterization of the population. All authors read and approved the final manuscript.

## Pre-publication history

The pre-publication history for this paper can be accessed here:


